# 

**Published:** 1894-01

**Authors:** 


### *MAGAZINES RECEIVED.*

### Lippincott's Magazine for January, 1894.

The complete novel in the January number of *Lippincott's* is "The Colonel," by Harry Willard French. Based on a romantic venture, in which the hero saves the heroine's life at sea, the tale goes on to study the characters of these two highly gifted idealists, and to trace the fortunes of mutual passion which neither is willing to own. The sentiment throughout is the purest and loftiest of which human nature is capable, and the chapters appeal to the reader's heart no less than to his brain.

Gilbert Parker supplies the opening chapter of a serial story, "The Trespasser," which will run through six numbers of the magazine. It deals with a Canadian of high family, who comes from a wild and wandering life to take his rightful place in England, and is of uncommon force and interest.

"Frenchy" is a domestic tale by Mollie Elliot Seawell. "A Mother and her Boy," by George Morley, is a pathetic sketch from every-day life.

"The Peninsula of Lower California," by James Knapp Reeve, gives valuable information concerning that little-known region, and corrects sundry errors of the "Encyclopædia Britannica" and other received accounts.

Mrs. Sherwood's "Recollections" of Rachel, Fanny Kemble, and Charlotte Cushman will interest many. Julian Hawthorne, in "A Poet of Manhood," pays tribute to the memory of Daniel L. Dawson.

Under the heading, "A Juvenile Revival," Thomas Chalmers celebrates the "Christian Endeavor" era. Frank Shelly writes of "Early Marriage Customs," and Charles Morris anticipates "The Twentieth Century." In "Talks with the Trade," F. M. B. answers some questions of young writers.

The poetry of the number is of unusual merit. It is by Martha T. Tyler, Celia A. Hayward, Kathleen R. Wheeler, Edward Oldham, M. S. Paden and the late Daniel L. Dawson.

